# A Review of the Nucleic Acid-Based Lateral Flow Assay for Detection of Breast Cancer from Circulating Biomarkers at a Point-of-Care in Low Income Countries

**DOI:** 10.3390/diagnostics12081973

**Published:** 2022-08-15

**Authors:** Busiswa Dyan, Palesa Pamela Seele, Amanda Skepu, Phumlane Selby Mdluli, Salerwe Mosebi, Nicole Remaliah Samantha Sibuyi

**Affiliations:** 1 Nanotechnology Innovation Centre, Health Platform, Mintek, 200 Malibongwe Drive, Randburg, Johannesburg 2194, South Africa; 2 Department of Life and Consumer Sciences, College of Agriculture and Environmental Sciences, University of South Africa, Private Bag X6, Florida, Johannesburg 1710, South Africa

**Keywords:** breast cancer, NABLFA, circulating biomarkers, point-of-care, low income countries

## Abstract

The current levels of breast cancer in African women have contributed to the high mortality rates among them. In South Africa, the incidence of breast cancer is also on the rise due to changes in behavioural and biological risk factors. Such low survival rates can be attributed to the late diagnosis of the disease due to a lack of access and the high costs of the current diagnostic tools. Breast cancer is asymptomatic at early stages, which is the best time to detect it and intervene to prevent high mortality rates. Proper risk assessment, campaigns, and access to adequate healthcare need to be prioritised among patients at an early stage. Early detection of breast cancer can significantly improve the survival rate of breast cancer patients, since therapeutic strategies are more effective at this stage. Early detection of breast cancer can be achieved by developing devices that are simple, sensitive, low-cost, and employed at point-of-care (POC), especially in low-income countries (LICs). Nucleic-acid-based lateral flow assays (NABLFAs) that combine molecular detection with the immunochemical visualisation principles, have recently emerged as tools for disease diagnosis, even for low biomarker concentrations. Detection of circulating genetic biomarkers in non-invasively collected biological fluids with NABLFAs presents an appealing and suitable method for POC testing in resource-limited regions and/or LICs. Diagnosis of breast cancer at an early stage will improve the survival rates of the patients. This review covers the analysis of the current state of NABLFA technologies used in developing countries to reduce the scourge of breast cancer.

## 1. Introduction

Globally, breast cancer is the second most prevalent cancer affecting women after cervical cancer. The number of diagnosed cases has increased rapidly, and 2.3 million cases are reported annually [1]. The majority (90–95%) of breast cancer cases are attributed to lifestyle, whereas 5–10% of these cases are heredity [2]. Strategies and campaigns have been developed to bring awareness and encourage women to screen and test for breast cancer. However, these are inaccessible to women in low and middle-income countries (LMCs), and as a result they are often diagnosed at an advanced stage when the disease can no longer be treated. Consequently, more than 685,000 mortalities are reported globally each year due to delayed diagnosis of breast cancer [3].

The breast cancer survival rate can be improved by early detection and therapeutic intervention. Currently, mammography, magnetic resonance imaging (MRI), X-ray imaging, ultrasound, CT scans, and tissue biopsies are the standard approved methods for breast cancer detection [4]. Despite their effectiveness, these methods have limitations in LICs, which include high cost, lack of trained personnel to operate and analyse data, and critically, a primary need for an invasive sampling procedure, such as a biopsy [5,6]. As such, countries with weak health infrastructure and limited screening or prevention programs continue to be affected by high mortality rates [7]. Therefore, there is a need to develop rapid and low-cost diagnostic tools that will be useful in LICs but still provide high sensitivity and specificity. Breast cancer diagnosis at the early stages is a crucial factor that will inform the disease treatment and management. Molecular changes during cancer development and progression are key to identifying individuals at risk, and it is critical to monitor these biomarkers, since this process could provide an effective way to follow the progress of breast cancer [8]. Cancer biomarkers are involved in various cellular processes that are essential for human life; any alteration in a biomarker’s level and expression could potentially serve as an indication for the development of cancer. Such biomarkers can be in the form of deoxyribonucleic acid (DNA), ribonucleic acid (RNA), proteins, metabolites [9], etc., and changes in their expression levels can be exploited to differentiate between healthy and infected individual [5].

Over the years, standard methods have been developed and used to detect or measure biomarker expression. These include enzyme-linked immunosorbent assays (ELISA) [10], radioimmunoassay (RIA) [11], and electrophoretic immunoassays [12]. These standard methods are known to give precise results, but their shortfall is that they require complicated equipment, multiple washing steps, and long turnaround times [13]. Therefore, the development of lateral flow biosensors (LFBs) for use at the POC has emerged as a feasible strategy for LMCs. Most importantly, LFB allows for the detection of circulating biomarkers in various biological fluids, such as saliva, blood, urine, serum, and plasma. Elevated levels of cell-free nucleic acids in the blood of cancer patients are amongst the biomarkers used to detect breast cancer [14] by ELISA and real-time polymerase chain reaction (RT or qPCR). However, these techniques are time-consuming and require costly instruments for detection, which becomes a limitation for use in LICs. The newly redesigned nucleic-acid based systems have shown potential for reducing the turnaround time. As a result, this review focuses on addressing the current trends in nucleic-acid-based lateral flow immunoassay systems for the detection of breast cancer.

## 2. Breast Cancer in Africa

Cancer has been reported to be the second major cause of death worldwide after heart-related diseases [15]. The most common cancers are breast, lung, prostate, cervix, brain, colon, thyroid, and pancreatic cancers [16]. Cervical cancer is the number one killer amongst women; 85% of cases and 88% of deaths occur in LMCs [17]. Even more concerning is that women in Sub-Saharan Africa (SSA) account for over a third of these cases, despite occupying only 14% of the female population in the world [18]. This region has also experienced a significant surge in incidence of breast cancer: from 19.7 per 100,000 to 36.9 per 100,000 in 2000 and 2015, respectively. In South Africa (SA) alone, breast cancer accounts for 22% of all malignancies [19], and it is predicted that 1 in 25 women are at risk of developing breast cancer in their lives [20].

Breast cancer survival rates vary globally, and high-income countries (HICs) have better survival than LMCs. For example, the United States of America (USA) has a survival rate of 89.9%, compared to 52.3% in SSA [21]. Most African countries have a five-year survival rate from the time of diagnosis to death/reoccurrence which is below 57%. For example, Kenya, Uganda, Malawi, and Nigeria had a 51.1% survival rate; compare that to New Zealand’s 90% survival rate [22]. The low survival rates in SSA are due to several factors, such as breast cancer diagnosis occurring at advanced clinical stages, co-morbid diseases, race, and aggressive pathological characteristics of breast cancer [23]. Global guidelines were developed to support program planning, implementation, monitoring, and early detection programs for breast cancer. These programs were found to be successful when tailored for specific communities and their needs, and by exploiting infrastructures and funding used for human immunodeficiency virus (HIV) programs [17]. Nonetheless, these programs are not accessible in LMCs. Furthermore, SSA countries are also burdened by other infectious diseases, further reducing the survival rates.

For instance, HIV/acquired immunodeficiency syndrome (AIDS) is one of the dominant infectious diseases in SSA; SA carries 20% of the global HIV burden, including 15% of new infections and 11% of AIDS-related deaths [23]. In 2018, about 7.52 million South Africans were reported to be living with HIV/AIDS, with 62% of them were on antiretroviral therapy [24]. Even though HIV is not an oncogenic disease, it can indirectly cause infected patients to develop other malignancies by suppressing T-cell function [25]. An estimated 30–40% of HIV patients are expected to have cancer in their lives [26]. HIV-positive patients usually present with advanced stages (stage III/IV) of breast cancer at diagnosis when compared to HIV-negative patients [20]. The survival rate of HIV/AIDS patients diagnosed with breast cancer is minimal [27]. This may be associated with the socioeconomic ills and inequalities that exist in SA, despite being rated by the World Bank as an upper-middle-income country in Africa [28]. Despite having multiple tertiary hospitals, state of the art oncology facilities [29], and national pathological laboratories with histopathological services, the survival rate in SA is still very low [30]. Private care patients are mostly funded by medical aid schemes, which only offer voluntary healthcare insurance to less than 15% of the population, whilst the majority of residents depend on public healthcare [31]. Women treated through private healthcare systems are likely to be treated by specialists with better oncological outcomes, whereas women cared by the public health system will only receive palliative attention instead of actual treatment. Additionally, the public healthcare system is burdened with high numbers [32], compounding issues related to late-stage diagnosis that are caused by inaccessibility to proper healthcare. The logistics and administration that must accompany diagnosis and treatment cause delays in therapeutic intervention and decrease the survival rate. The issues include (1) long-distance travel to hospitals and at times requiring visits to more than two healthcare facilities for diagnosis and treatment [33], (2) a patient needing a referral from a healthcare centre prior to attendance by a nearby hospital [34], and (3) the lengthy and costly diagnosis procedure. The procedure for diagnosis involves clinical examination and staging; imaging with mammography and ultrasonography; an image-guided core needle biopsy for histological diagnostic confirmation; tumour grading and receptor subtyping, which are performed by the national pathology laboratories [32]. The results take two to three weeks to obtain. This lengthy waiting period discourages the patients from returning for their results, as this will invariably involve a cost. The aforementioned reasons could be some of the reasons that women of African origin have the lowest registered cases of breast cancer [35]. Though contradictorily, the number of women diagnosed with breast cancer is now increasing due to the behavioural and biological risk factors [23]. Other reasons include illiteracy, health beliefs, policy constraints, and social-cultural factors [36]. Therefore, a screening device at a point-of-care for breast cancer has the potential to save lives and lessen the burden on the SA healthcare system [37] and the other LICs.

## 3. Breast Cancer Symptoms and Diagnosis

Breast cancer is a type of cancer that occurs mainly in women, though a small percentage of men are affected [38]. The cancerous cells form a tumour or a lump in the breast that can be felt or visualised through an X-ray. Like other cancers, breast cancer can invade and spread to other tissues surrounding the breast and other parts of the body [5], such as bones, the liver, lungs, or the brain [39]. It can be classified as invasive or non-invasive. In an invasive breast cancer, the cancerous cells spread to the ducts and possibly to the lymph nodes. Conversely, non-invasive cancer is confined to ducts or lobules where the cancer originated [40].

Early breast cancer usually does not cause pain or show any noticeable symptoms, and can go unnoticed for years. As the cancer progresses, early signs and symptoms can be physically observed: a lump or thickening in or near the breast; a change in the size or shape of the breast; nipple discharge, tenderness or retraction (turning inward); skin irritation, dimpling or scanlines [41]. These changes are not breast-cancer-specific and can occur as part of different conditions, such as fribrocystic [42] and mastitis cystic breast disease [43]. Nevertheless, having one or more of these symptoms can raise health concerns for breast cancer [39].

### 3.1. Breast Cancer Susceptibility Genes

Breast cancer occurs because of the genetic modifications or mutations in normal breast cells. Some mutations significantly increase the risks of certain cancers. DNA mutations linked to breast cancer are hereditary, whereas some are acquired [44]. Hereditary breast cancers usually occur earlier in life than the acquired (sporadic) cases, and are more likely to involve both breasts [45]. It is estimated that 5% to 10% of all hereditary breast cancers are due to known breast cancer susceptibility genes [46]. These genes are divided into low, moderate, and high-risk breast cancer susceptibility genes (Table 1). The high-risk breast cancer susceptibility genes include *breast cancer 1* (*BRCA1*), *BRCA2*, *phosphatase and tensin homolog* (*PTEN*), *tumour protein p53* (*TP53*), *serine/threonine kinase 11* (*STK*), and *CDHI*. Checkpoint kinase *1* (*CHEK1*), transforming growth factor *β1 (TGF*-*β1)*, caspase (CASP)8, and Ataxia telangiectasia mutated (*ATM*) genes belong to the low to moderate-risk breast cancer susceptibility genes [45].

*BRAC1* and *BRAC2* are high-risk genes with 59–87% and 35–80% chances of developing cancer, respectively. *BRAC1* and *BRAC2* mutations are responsible for more deadly tumours and are located on chromosome 17 and chromosome 13, respectively. *BRAC1* has 300 mutations that cause cancer, and *BRAC2* has 1600 [47]. Some of the mutations include 185delAG; 6174delT; 5382ins; CS1832P; T2766I; N2781I; and K2860T, K3083E, or 9475A > G. These mutations are found to be more common among certain geographic or ethnic groups; for example, *BRAC1* (3036del4) and *BRAC2* mutations are high in Jewish women from Ashkenazi (Eastern Europe) [48]. Asian, Hispanic, and Native American women are at a lower risk of carrying breast cancer susceptibility genes [49], whereas in SA, *BRCA1*, *PALB2* and *RAD5IC* genes are often responsible for breast cancer diagnosis [50]. Women who have inherited some of these genetic mutations have a high risk of developing breast cancer, ovarian cancer, colon cancer, and other types of cancer during their lives. Men with *BRCA1* mutations (3232A>G) also have an increased risk of developing breast cancer [51]. *BRCA1* mutations are also associated with increased risks of other cancers; for example, pancreatic cancer, prostate cancer, and ovarian cancer [52]. Similarly, mutations in the *BRCA2* gene are also associated with increased chances of developing male breast cancer and cancers of the prostate and pancreas. An aggressive form of skin cancer (melanoma) is also more common amongst people who have BRCA2 mutations [53]. Identification of these mutations has been a crucial breakthrough in the research and development of more specific and selective diagnostic tools.

### 3.2. Diagnosis of Breast Cancer

Breast cancer screening and diagnosis starts with self-examination, which is performed lying down or standing, placing the right arm behind the head. The left hand is used to feel for lumps by using an over-lapping dime-sized circular motion of the finger to touch the breast for any lumps [54]. The screening can be followed by clinical validation. The current diagnostic methods used for breast cancer are shown in Table 2. These methods include biopsy, endoscopy, diagnostic imaging methods, and mammography. In biopsies, a small tissue sample is surgically removed from the suspicious growth area of the breast and examined under a microscope for the presence of cancer cells. This procedure can be performed by a surgeon or a radiologist [55]. Endoscopy involves the insertion of a flexible plastic tube with a tiny camera at the end into the nipple through the breast ducts, deep into the breast. This tube allows a physician to view the lining of the lactiferous ducts and look for abnormal tissue [54]. Diagnostic imaging methods such as X-ray imagine, computerized axial tomography (CAT), magnetic resonance imaging (MRI), and ultrasound are image-based methods for studying the anatomy of the breast and can identify any possible abnormalities [56,57]. Ultrasound evaluates whether the breast is filled with fluid (a cyst) or solid objects (tumours) [58]. The advantages and disadvantages of these diagnostic tests are summarized in Table 2. Most of these methods are invasive, time-consuming, require skilled personnel, use expensive equipment, have long turnaround times, and most importantly, are costly. The limitations of physical and image-based methods for breast cancer diagnosis [56] could be overcome by the use of molecular methods that detect disease biomarkers, such as immunoblotting, immunohistochemistry (IHC), enzyme-linked immunosorbent assay (ELISA), and in-situ fluorescence hybridization (FISH) [59]. Advancements in research and technology have enabled researchers to come up with improved molecular methods for diagnosis. The latest addition to breast cancer diagnostics is the Prosigna assay, which was approved by the FDA to determine the risk of recurrence in breast cancer patients after surgery. The assay studies changes in mRNA expression of a panel of 50 genes associated with various molecular subtypes of breast cancer, collectively known as the Prediction Analysis of Microarray 50 (PAM50) [60,61,62]. Other new and emerging preclinical assays that are being explored for diagnosis include electrochemical technologies such as electrochemical biosensors, nano-transistors, photonic crystals, and microfluidics-based technologies [63]. Although all these technologies provide improved diagnostic methods, they are not suitable for use in LMCs, as they require expensive equipment, sample pre-treatment, and trained personnel [60,63]. Thus, there is an urgent need for a quick breast cancer diagnostic method or technique that will be minimally invasive, rapid, and less expensive [54].

## 4. Nucleic Acids in Breast Cancer Diagnosis

Body fluids such as blood, urine, and cerebrospinal fluid contain the blueprint that can reflect the health status of an individual. Blood has been ubiquitously used over the years to diagnose various diseases. An average human adult has about five litres of continuously circulating blood that delivers nutrients and transports metabolic waste throughout the body [65]. Blood is made up of 54.3% plasma, 45% red blood cells, and 0.7% white blood cells by volume [66]. Plasma, the fluid part of blood, consists of proteins, nucleic acids, nutrients, and waste products. It also maintains the electrolyte balance and protects the body from infection and blood disorders [67]. Serum, obtained after blood clotting [54], tends to be used for detection of biological molecules present in the blood. Nucleic acids are some of the circulating biomarkers found in blood, and possibly other easily accessible biological fluids; thus, nucleic acids can be used to differentiate between healthy and disease states in diagnostics [68].

### 4.1. Circulating Biomarkers for Breast Cancer Diagnosis

In infectious diseases, nucleic acids offer additional advantages as a type of biomarker over antigens, antibodies, and metabolites. Specificity is one such advantage, as bacterial and viral DNA that are shed in the host during infection can be distinguished from the hosts’ DNA and microbial strains. These discriminating features are not always expressed at the phenotypic level; for instance, severe acute respiratory syndrome corona virus (SARS-CoV-1) and SARS-CoV-2 spike proteins can be differentiated via DNA rather than protein. Additionally, DNA, unlike antibodies, is detected almost immediately after infection, which means that a disease can be timeously diagnosed without requiring further incubation [69].

Nucleic acids are carriers of genetic information and can be secreted into the bloodstream in the form of circulating nucleic acids. They are detectable in small amounts in the sera of healthy individuals [70], which means elevated levels would suggest epigenetic alterations of a primary tumour [71]. It has been reported that the circulating nucleic acids are released into the bloodstream by proliferating or dying (both necrotic and apoptotic) cells [72], and also carried by exosomes shed in body fluids. Thus, exosomes can also be targeted as biomarker reservoirs, as they contain cellular components derived from their parental cells [73]. The circulating tumour DNA (ctDNA) and microRNA (miRNA) found in serum are known to contain tumour-specific mutations [65]. These molecular biomarkers are released in the blood during cancer growth and progression [70], and their expression repertoire can be used to detect and classify cancer stages or prognoses. From this, better therapeutic and diagnostic strategies can therefore be derived and implemented [74].

DNA in the human bloodstream was first reported in 1948 [75]. Attention was drawn to these findings in 1966 when the presence of DNA was reported in serum from patients suffering from systemic lupus erythematosus. Since then, DNA has also been detected in patients with other diseases, such as hepatitis, metastatic carcinoma, and miliary tuberculosis; and it was proposed that these DNA molecules originate from endogenous tissue breakdown [76]. Several years later, the presence of DNA in the sera of cancer patients with various cancers, such as breast, lung, cervical, ovary, and lymphosarcoma cancers, was detected using RIA. The amounts of DNA in these samples were quantified based on their affinity for DNA antibodies produced from patients with lupus erythematosus. High levels of DNA were detected in 50% of sera from cancer patients, of whom the majority were metastatic cancer patients [77]. Stroun et al. reported that the increased ctDNA content exhibited genetically identical characteristics to tumour DNA [65]. Two groups also confirmed the presence of tumour-associated oncogenes, namely, BCR ABL [78] and CA19.9 [79], for leukaemia and pancreatic cancer, respectively.

The discovery of DNA in the blood led researchers to find other types of blood circulating nucleic acids. RNA was also found to be one of the nucleic acids secreted in plasma in the form of microRNAs (miRNA) [68]. Elevated levels of microRNAs were also found in the blood samples from breast cancer patients, and were confirmed to be associated with tumour development and progression. The circulating concentrations of miRNAs, particularly miR-10b, miR-34a, miR-141, and miR-155, were higher in patients with primary breast cancer than in patients with no cancer [80]. Elevated levels of miR-885-5p, miR-1, miR-95, and miR-929, were also reported in blood from patients with breast cancer [81]. Iorio et al. also identified 13 miRNAs, which included miR-21, miR-125a, miR-205, miR-335, and miR-126, which were secreted into the bloodstream of breast cancer patients, and this revealed valuable biological information about the tumour [82]. These reports provided evidence that breast cancer cells do secrete cancer-related nucleic acids (DNA and miRNAs) which can be detected in the bloodstream.

### 4.2. PCR-Based Diagnostic Methods for Detection of Nucleic Acids

Molecular techniques such as PCR, ELISA, IHC, FISH, and mass spectrometry (MS) [83] have been instrumental in the detection of genetic mutations caused by pathogens. These tests are used for molecular diagnoses of diseases from various type of samples, including blood [74]. PCR-based techniques are by far the most reliable and most sensitive tests. The various methods are summarised in Table 3 below, including their disadvantages, which signify a need for cost-effective, easy to use, and rapid test devices.

## 5. NABLFA for Rapid Diagnostics

**Figure 1:**
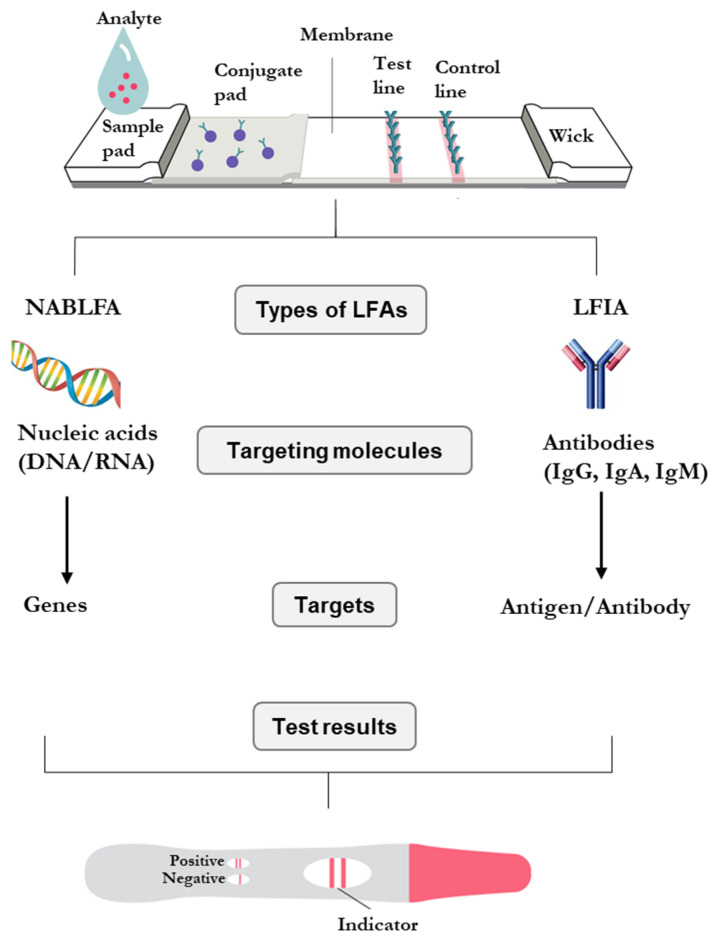
Types of clinically used LFAs for detection of nucleic acids (NABLFA) and antibodies (LFIA) found in biological fluids [90].

LFAs are based on the detection of analyte/antigens in body fluids and have emerged as reliable techniques for the diagnosis of several diseases [89]. There are two types of lateral flow formats, namely, the antibody and nucleic acid-based LFAs, as shown in Figure 1 [90]. The sandwich LFAs are used to detect antigens with multiple epitopes, such as those used to detect infectious diseases. The competitive LFAs test for antigens with single epitopes; examples are drug abuse tests [91]. LFAs are cost-effective and rapid compared to the molecular tests, with turnaround times of 10–15 min. Although the immuno-based assays (lateral flow immunoassay, LFIA) have been successful in diagnostics for decades, they have some limitations: their sensitivity relies on the concentration of the test specimen, and the test samples must be in solution [92]. The sensitivity of LFAs was shown to be improved from the μM level in LFIA to the aM level in systems that incorporate a pre-amplification step for test samples in NABLFAs. The sensitivity of an NABLFA is comparable to those of molecular tests and immunoassays [93].

### 5.1. NABLFA

NABLFAs are under development for the detection of various genetic markers (DNA, RNA, or miRNA) that are specific for infectious and chronic diseases [94]. The NABLFAs have the added advantage of amplifying the nucleic acid targets that are specific to the analyte, as such the concentration can be enriched and detected [95]. NABLFA gives high sensitivity and specificity, similar to that of molecular tests, compared to the antibody-based LFAs [96].

Nucleic acid-based tests are essential in the diagnosis of genetic diseases. Several NABLFAs have been successfully developed to detect DNA, mRNA, proteins, and other biological agents [97]. Developing these nucleic-acid-based tests for fundamental research and clinical applications has become widely attractive because they offer simplicity, and are less time-consuming and labour intensive compared to conventional PCR methods [98,99]. Most NABLFAs are based on binding of hapten to target molecules, such as antibody or protein, in the test sample. The target molecules are first amplified using hapten-labelled primers [99]. The NABFLA follows the same principles as LFAs, except that the detection procedure starts with the amplification of genes of interest using PCR and use of the PCR product (amplicons) as a test sample [100]. Colorimetric detection is also an integral part of NABLFA which offers a more straightforward option to detect or identify PCR products by the naked eye, without additional equipment or the need for skilled personnel. NABLFAs are favourable diagnostic devices due to their ease of use [94]. The NABLFA follows in the success of nucleic acid amplification technologies (NAATs) [101], and unlike NAATs, the end-point test uses a rapid and POC system that can be used in scarcely resourced settings. The NABLFAs have been used to detect nucleic acids in food pathogens [91], infectious diseases [102], and cancer biomarkers [89].

### 5.2. NABLFA in Cancer Diagnosis

**Figure 2:**
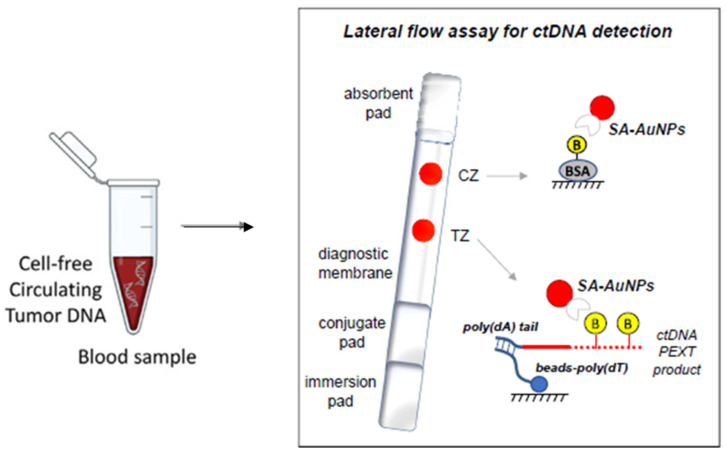
AuNPs-based NABLFA for rapid detection of ctDNA in CRC blood samples. Adapted with permission from [103]. 2021, Elsevier.

The feasibility of NABLFA has been demonstrated in the detection of various cancers [89]. Notably, a group in Greece developed an NABLFA for the detection of Kirsten rat sarcoma viral oncogene homologue (KRAS) mutations in DNA samples extracted from colorectal cancer (CRC) cells and blood [103]. The NABLFA was developed for the analysis of blood samples from CRC patients, wherein four single nucleotide polymorphisms (SNPs) that matched the normal KRAS gene and three of the most common mutations in the KRAS gene correlating to CRC in synthetic DNA samples, cancer cells, and ctDNA were detected [103]. *KRAS* is commonly used for cancer prognosis, response to chemotherapy, and resistance to anti-EGFR therapy [104], and its presence in blood samples could serve as a theragnostic biomarker. The gold nanoparticles (AuNPs)-based NABLFA (Figure 2) had high specificity and was able to differentiate single KRAS mutations in ctDNA extracted from cells and blood. This system demonstrated that the existing biomarkers present in low amounts can be detected in various diseases, including breast cancer [103]. Although the amplification step is crucial in cases where the biomarker is present in undetectable or low levels, it becomes a limitation for low resource settings and is unsuited for a POC testing. Therefore, there is still a need to develop less expensive diagnostic methods that can lead to rapid detection of breast cancer [54] at a POC to accommodate the LMCs.

Next-generation PCR technologies that involve rapid amplification and detection of DNA and have a potential use in LMCs were devised, such as continuous flow PCR, droplet PCR, digital PCR, ultrafast photonic PCR, and insulated isothermal PCR [105]. However, temperature control in these devices still proves to be a limiting factor; hence, isothermal amplification methods such as loop-mediated isothermal amplification (LAMP) are better constructed for in-field use [106]. Despite the existence of other isothermal methods, LAMP has stood out amongst the other amplification assays, which include the strand displacement amplification, helicase-dependent amplification, rolling circle amplification, recombinase polymerase amplification, and nicking enzyme amplification reaction (NEAR) [107]. LAMP is considered superior due to several reasons: (1) LAMP is inhibitor-tolerant—that is, amplification is not suppressed by biologically-borne inhibitors found in blood, urine, and saliva; (2) amplification can be readily achieved from unprocessed raw samples, such as swabs and whole blood; (3) the prolonged storage time of reagents that have been preserved by lyophilisation; and lastly, (4) the protocols for this method are readily accessible, and its patent is not as restrictive as the NEAR and recombinase polymerase amplification assays [106,107].

In the year 2000, Notomi et al., discovered a PCR-inspired LAMP method. Amongst the many features of this isothermal method that make it favourable, is the use of the high strand displacement Bst polymerase, which operates under isothermal conditions. This transcends the limitations of using complex and expensive heating instruments that are needed for denaturing DNA [107,108]. As this method gained popularity, different and improved versions of it evolved—namely, the reverse transcription LAMP (RT-LAMP), which is the most widely used, the multiplex LAMP, electric LAMP, and in-disc LAMP [108]. The relatively high specificity of this technique has been attributed to the four (or six) primers that recognise and bind to six (or eight) different sites of the target DNA [109]. Once amplification of the targeted DNA is completed, the amplicon needs to be detected and/or quantified. LAMP is a highly flexible assay that accommodates different detection methods, such as turbidimetric, fluorescent, chemiluminescent, electrochemical, and colorimetric detection [107]. LAMP was then integrated with other technologies for rapid diagnosis of disease biomarkers. A LAMP-based microfluidic device that incorporates the nucleic acid extraction, amplification, and LFA steps in a single device demonstrated the feasibility of these systems for use at POC [110].

Clustered regularly interspaced short palindromic repeats (CRISPR), which are found in bacteria and archaea, confer acquired immunity against foreign genetic material, such as bacteriophages and plasmids. These hypervariable CRISPR are able to take up and store the foreign genetic material to form short DNA sequences. Ultimately, these sequences are recognised and hydrolysed by caspases, forming part of the basic concept of the CRISP–Cas based diagnostic assays [111]. Efforts to detect circulating nucleic acids using amplification-free assays were reported. This assay used oligonucleotide-templated LFAs to detect circulating microRNAs in blood samples [112].

### 5.3. NABLFA in Breast Cancer Diagnosis

**Figure 3:**
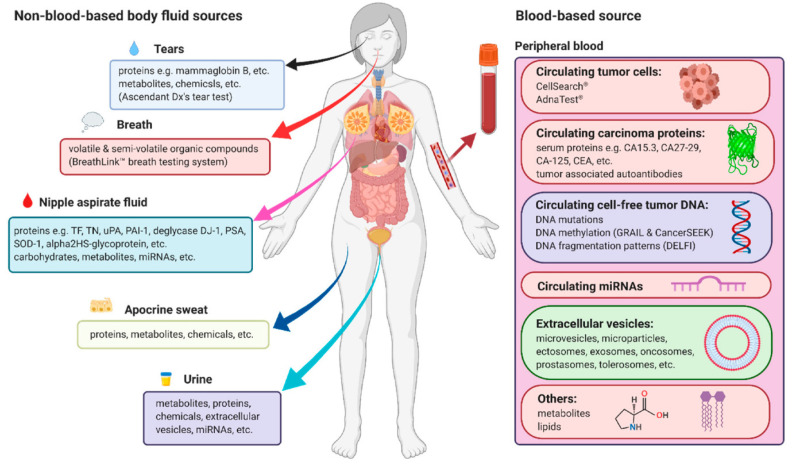
Non-invasive sampling of biomarkers that can be used for early detection of breast cancer in human samples [68].

NAATs have played a huge role in the development of molecular tests that are highly sensitive and capable of studying genes associated with various diseases and their diagnosis, including breast cancer. PCR-based assays and FISH are still used to evaluate the expression of BRCA and HER2/neu genes in breast tissues, among others. Using a one-step nucleic acid amplification assay, forkhead box P3 and cluster of differentiation 4 genes were detected in sentinel lymph nodes samples from metastatic breast cancer patients [113]. However, tissue and liquid biopsy sampling are highly invasive, which led to the use of less invasive sampling methods. Circulating biomarkers associated with breast cancer were found in various body fluids, such as blood [80,81,82], urine [114], nipple aspirate fluid, tears, and sweat [68] (Figure 3); it is pending validation for clinical application. The non-invasive biomarkers, their sources, and tests used in their detection have been reviewed elsewhere [68]. Most of these biomarkers are measured in the body fluids using molecular tests, and in recent years, these techniques have been evolving into systems that can be used at the POC and in low-resource settings. The proposed NABLFA diagnostic test is user friendly and offers rapid detection of target biomarkers in solution. The NABLFA is compatible with the current screening or molecular diagnostic tests for disease biomarkers and presents a cost-effective system for LMCs. Thus, the NABLFA is of clinical value and displays features that are desirable for POC testing even in resource-limited settings. The lack of clinical NABLFAs or LFAs for diagnosis of cancer does not discredit their value; in fact, they could tap into the same success and market as the LFAs for infectious diseases [115], and their convenience can encourage patients to do regular check-ups. The LFAs are currently being integrated for cancer diagnostics; this was brought to light by the collaborative effort between SCIENION (Berlin, Germany) and the Institute for Prevention and Occupational Medicine of the German Social Accident Insurance (North Rhine-Westphalia, Germany) to develop a bladder cancer LFA. The multiplex LFA will be used to detect ten bladder cancer biomarkers in urine samples at a POC [116], further validating the usefulness of these systems in LICs. Although NABLFAs have the potential for improving testing accessibility and clinical outcomes, the technology is novel and has limitations. Less than 20% of healthcare facilities in seven Sub-Saharan countries have the capacity for deploying this technology, compared to 80% coverage in HICs. The limitations have been attributed to minimal operational education, high cost, and limited technological options [117]. Additionally, the POC device has to be functional under various environmental conditions. Similarly to TB testing, only a third of newly infected patients were diagnosed using LFA, and these are some of factors that cause resistance toward new technologies. When the LFIA-based TB LAM Alere Determine™ was initially introduced in South Africa, the uptake was very slow, owing to lack of proper establishment of procurement processes, low confidence in use of the device, and inaccessibility in some areas. Since its rollout, the detection of TB cases more than quadrupled between 2017 and 2020 [118]. Thus, with much persistence through education, acquiring resources, and increasing capacity, the prospects for NABLFAs in LICs are encouraging.

## 6. Conclusions

Early diagnosis is a vital part of treating and managing breast cancer and ensuring the higher rates of patient survival. Although NAATs have maintained a significant role in disease diagnosis, the systems used are not yet suitable for a POC testing, since nucleic extraction and the amplification steps are crucial for successfully developing a NABLFA with robust sensitivity that is suitable for POC testing. This challenge can be overcome by using technologies that incorporate the extraction and amplification processes as a single step, which ultimately requires limited use of instrumentation. This is true of isothermal techniques such as the RT-LAMP, which require no thermo-cycler. Thus, RT-LAMP can be combined with an LFA [119]. In the last few years, LFAs have been adopted for the detection of genetic markers in solution, and advanced into systems that can detect circulating molecules in biological fluids without the need for an instrument. Colorimetric LFAs are user friendly, and any person can use and interpret the results with ease. The presence of an analyte is reflected in a coloured line that can be visually detected with the naked eye. The popularity of LFAs is based on their simplicity and the enabled rapid diagnoses of diseases at POC [120]. As such, NABLFAs could overcome limitations that are associated with breast cancer diagnosis in LICs/LMCS. Circulating Breast cancer biomarkers have been identified, and they could be instrumental in the development of LFAs that do not require skilled personnel or a controlled environment. Moreover, these biomarkers can be detected in easy-to-access samples, such as saliva, urine, and sweat [68]. The LFA market is predicted to reach 12.6 billion USD in 2026, demonstrating the importance and uptake of these technologies by clinical practitioners [115]. Various companies have introduced various NABLFA for POC testing for cancer biomarkers [89]. Although these tests are not yet available for breast cancer, the studies under review serve as proof of the notion that they can be a reality for all cancers, including breast cancer.

## Figures and Tables

**Table 1:** List of known breast cancer genes.

Low-Risk Genes	Moderate Risk Genes	High-Risk Genes
*Fibroblast growth factor receptor 2*	*CHEK2*	*BRCA1*
*TOX high mobility group box family member 3*	*Partner and localizer of BRCA (PALB) 2*	*BRCA2*
*mitogen-activated protein kinase kinase kinase 1*	*BRCA1 Interacting Protein 1*	*PTEN*
*FAM84B/C-MYC*	*ATM*	*TP53*
*lymphocyte-specific protein 1*		*STK11*
*NIMA Related Kinase 10*		*CDHI*
*Cytochrome c oxidase 11*		
*CASP8(D302H)*		
*TNP/IGFBP5/IGFBP2/TNSI*		
*Neurogenic locus notch homolog protein 2/Fc gamma receptor Ib*		
*RAD5ILI*		
*mitochondrial ribosomal protein S30/FcgammaRI*		
*Estrogen receptor I*		

**Table 2:** Advantages and disadvantages of different techniques for the diagnosis of breast cancer.

Technique	Advantage	Disadvantage	* Cost Per Consultation (ZAR)
Biopsy	The results provide all the characteristics of the cancer cells.	Require surgery to get a sample women who may not have breast cancer will have the surgery just to clear them.	R11,000–R26,000
Endoscopy	More details about the cancer cells (colour, texture). Short operation time Can see cancer in hidden areas.	Requires surgery and may leave a scar.	R1000–R4000
Diagnostic Imaging (CAT, X-rays, MRI)	Screening of high-risk woman gives more information about suspicious area. Detect the spreading of cancer to other parts of the body and Monitor reoccurrence after treatment.	A contrast solution (dye) is intravenously injected into your arm. This dye can affect your kidneys, a test for kidneys must be performed before it’s injected. The procedure is invasive and requires too many tests.	R6000–R12,000
Breast self-exam	Detect tumour at an early stage.	Validation must be followed-up with molecular tests.	Free

* Note: The costs for consultation in South Africa were adapted from [64]. 2021, Mediclinic tariffs.

**Table 3:** Clinical and pre-clinical molecular methods for detection of nucleic acids.

Method	Advantage	Disadvantage	Ref.
Microarrays	Analysis of thousands of genes in a single test to create molecular tumour profiles	Require long hybridisation times Prolonged wash steps that can take up to 24 h	[84]
RT-PCR	DNA amplification increases sensitivity Test multiple samples simultaneously	Requires a series of temperature changes Tedious sample preparation Equipment	[85]
Nano pore sensor	Label-free. Small sample size Amplification free Distinguish single-nucleotide differences	No reproducibility or adaptability of biological system	[86]
Micro-fluid devices	Rapid purification of nucleic acids	Challenging to integrate blood pre-treatment steps	[87]
A three-mode electrochemical sensor (HPD-SENS)	Detect low concentrations of miRNA 10 aM to 1 mM range Multiple miRNAs on a single electrode. Exhibits high selectivity and specificity.	Detection of low of copy number of sample of DNA/RNA in samples for early onset of a disease	[88]

## Data Availability

Not applicable.
